# Limited capital? Explaining transatlantic divergences in artificial intelligence startup financing

**DOI:** 10.1371/journal.pone.0342837

**Published:** 2026-11-05

**Authors:** Christian Hermann Hennings, Dirk Schiereck

**Affiliations:** Technical University Darmstadt, Darmstadt, Germany; Gachon University, KOREA, REPUBLIC OF

## Abstract

Artificial intelligence (AI) is a global general-purpose technology (GPT), yet the financial systems that scale AI ventures differ markedly across regions. This paper compares AI-focused venture capital (VC) dynamics in Europe and the United States from 2000 to 2025 using approximately 8,000 AI-related VC rounds from PitchBook and macroeconomic and institutional data from the World Bank. Exploiting the 2021 generative-AI inflection point as a common technological shock that triggered a structural reallocation of venture capital toward AI ventures, we apply a difference-in-differences (DiD) design to distinguish between startup formation and scale-up financing. Descriptive evidence shows that Europe keeps pace with the US in AI deal counts, suggesting no major pipeline gap in startup formation. However, Europe lags substantially in AI investment volumes and in AI’s share of total VC funding. In the Post-period, AI accounts for around 60% of VC investment volume in the US, compared to roughly 31% in Europe. Difference-in-differences estimates are consistent with parallel trajectories in AI deal activity but point to large and statistically significant transatlantic gaps in AI investment volume and investment intensity. These results are robust to macroeconomic and governance controls, a formal event-study test of pre-treatment trends, and a sensitivity analysis that excludes the United Kingdom (which alone accounts for roughly 31% of European VC volume), a placebo test using a false treatment year, and the exclusion of the COVID-19 period. Interpreting the findings through GPT diffusion and the Varieties of Capitalism framework, we interpret the pattern as being consistent with the US liberal market economy having a structural advantage in mobilizing large-scale risk capital. The results inform current debates on European technological sovereignty, the Capital Markets Union, and AI-related policy initiatives.

## 1 Introduction

Artificial intelligence (AI) has evolved rapidly from a niche research field into a foundational technology with economy-wide implications. Recent work by Calvino et al. [1] and the OECD’s AI program suggests that generative AI should be classified as a general-purpose technology (GPT), comparable to past breakthroughs like electricity and the microprocessor. According to classic GPT theory [2], the greatest productivity gains from AI do not come from the invention itself but from waves of complementary innovation throughout the economy, which are highly capital-intensive.

Venture capital (VC) is central to financing these waves of complementary innovation. VC investors screen high-risk projects, provide funding, and monitor portfolio firms to enhance innovation outcomes [3]. According to recent industry reports, more than half of all VC deals globally now involve AI-related firms [4,5]. However, this boom is geographically uneven: the United States (US) attracts most of the global AI VC activity, while Europe captures only a modest share despite its strong research base and active startup ecosystem [4–7].

This apparent “innovation paradox,” Europe sitting on a “goldmine” of ideas but lacking impact [8], reflects broader concerns about a gap in European scale-up financing [9]. Reports by the European Investment Bank [10], the European Investment Fund [11], the European Commission [7,12] and the IMF [13] all describe European startups as subsidy-rich but capital-poor [14] and as facing structural obstacles when raising large growth rounds. Recent policy debates around the EU AI Act point to the tension between Europe’s ambition to lead in trustworthy AI and the risk that regulatory complexity and underdeveloped capital markets could lead to a reliance on foreign platforms, primarily those in the US.

This paper addresses one question: Is the transatlantic gap in AI venture capital investments primarily a problem of innovation or a problem of finance? In other words, does Europe lack promising AI ventures, or does it lack the financial infrastructure needed to develop them into global leaders? Using the Varieties of Capitalism (VoC) framework [15], we view the US as a prototypical liberal market economy (LME) with deep, equity-based capital markets and Europe as a group of coordinated market economies (CMEs) with bank-centric, more risk-averse financial systems [16,17].

We empirically construct a regional-level panel of AI VC activity, covering Europe and the US from 2000 to 2025. While the academic foundations of the modern AI boom date back to the 2012 deep learning breakthrough [18–22], the reallocation of venture capital toward AI is a distinct and later event. It is useful to separate two moments: the onset of the generative-AI investment cycle in 2021, when the first commercial large-model APIs and an initial wave of generative-AI VC fundraising appeared, and the mass-market consumer inflection associated with the release of ChatGPT in late 2022. Because our treatment concerns the reallocation of venture capital, an investment decision by funds rather than a consumer-adoption event, we anchor it on the 2021 onset: we use Q2 2021 as the start of the treatment window and Q3 2024 as the start of a “post”-period that traces the consolidation of AI as the dominant theme in venture portfolios. As shown in Section 3 and S7 Table, our central result is robust to placing the break instead at the Q4 2022 ChatGPT release. Using a difference-in-differences (DiD) design, we evaluate whether the US ecosystem reallocated capital toward AI more strongly than Europe did.

We report three main findings. First, Europe kept pace with the US in AI deal counts after the treatment break, with no collapse in startup formation. Second, the US experienced a disproportionate surge in AI investment volumes and AI’s share of total VC, resulting in a significant financing advantage. In the Post-period, AI accounted for approximately 60% of US VC volume, whereas in Europe, it accounted for only about 31%. Third, these patterns remain robust when controlling for macroeconomic variables, innovation inputs and governance indicators, and when trimming the largest deals.

The paper makes three contributions. First, we integrate GPT theory, VoC and recent work on AI VC to frame the AI boom as a stress test of regional financial systems. Second, we present one of the first DiD analyses of AI VC at the regional level, using a long panel spanning both the build-up of the deep learning research base from the early 2010s and the venture-capital reallocation triggered by generative AI from 2021 onward. In terms of policy, we derive implications for Europe’s Capital Markets Union, the EU AI Act, and emerging European fund-of-funds initiatives.

The rest of the paper is structured as follows. Section 2 develops the conceptual background. Section 3 describes the data and methods. Section 4 presents the empirical results, including robustness checks. Section 5 discusses the findings in the context of existing literature and policy debates. Section 6 concludes.

## 2 Theoretical background

### 2.1 Artificial intelligence as a general-purpose technology and finance

GPTs are characterized by three properties: pervasiveness across sectors, continuous improvement, and the ability to generate complementary innovations [2]. GPT diffusion typically unfolds over decades and requires substantial investments in complementary assets, including skills, infrastructure, and organizational changes. Countries with flexible, well-developed financial systems can redirect resources toward new technologies more quickly, allowing them to reap greater benefits from GPTs [2,23].

Recent OECD work argues that generative AI now meets these GPT criteria [1,4]. The impact of AI depends heavily on firms’ ability to experiment with new business models and integrate AI into production and services. These activities are risky, intangible-intensive, and often pre-profit, making them difficult to finance through traditional bank lending [24–26]. VC, with its tolerance for uncertainty, staged financing, and governance role, is therefore a key mechanism for reallocating capital to AI experiments [3,27].

Complementary investments for GPT diffusion include not only data infrastructure and skills but also organizational capabilities to experiment with and adopt frontier tools. Recent protocol-based work focusing on startups’ adoption of ChatGPT proposes an extended technology-adoption framework (building on TAM) and explicitly frames adoption as an experimentation-driven process shaped by perceived benefits and barriers from the entrepreneur’s perspective [28].

Montanaro et al. [29] show that VC investments in AI ventures differ systematically from investments in other Industry 4.0 startups. AI deals tend to be smaller but more sensitive to factors such as venture stage, country-level AI development and investor experience. These findings suggest that the institutional context is important for allocating AI capital and that regions with more developed late-stage VC markets may be better positioned to support the capital-intensive phases of GPT diffusion.

### 2.2 Varieties of capitalism and venture capital

The VoC framework [15,30] distinguishes between LMEs and CMEs. LMEs, such as the US, rely heavily on competitive markets for finance and labor. In contrast, CMEs, which are prevalent in continental Europe and the Nordic countries, depend more on relational coordination and bank-based financing.

VC naturally fits into LME environments. Black and Gilson [27] argue that a thriving VC industry requires an active stock market because IPOs provide high-return exits that justify early-stage risk-taking. Jeng and Wells [23] and Palomino et al. [16] find that IPO activity, market capitalization and private pension funds are key determinants of national VC intensity. Grilli et al. [17] emphasize the importance of legal investor protection, taxation and labor market institutions. Dimitrova and Eswar [31] demonstrate that higher capital gains taxes reduce VC activity and startup innovation. These findings show how sensitive VC is to policy design.

In CMEs, large banks and corporate insiders play a more prominent role in financing innovation. While this can support incremental innovation and long-term relationships, it is less suited to funding highly uncertain, disruptive ventures [32–34]. The EIB [10] and EIF [11] reports note that, despite strong local success stories, Europe’s VC ecosystem remains smaller, more fragmented, and more dependent on public actors than its US counterpart [13,35,36].

### 2.3 The European scale-up finance gap and artificial intelligence

A substantial literature examines whether and how access to finance for high-growth firms differs across regions, with particular attention to firms’ ability to scale beyond early development stages. A recurring finding is that while innovative European startups frequently succeed in attracting seed and early-stage funding, raising large growth-stage rounds appears to be more difficult on average than in the United States [9,16,17,23,37–39]. At the same time, the literature remains divided on the underlying mechanisms and on whether observed differences reflect binding financial constraints, firm heterogeneity, or broader institutional features.

On the supply side, numerous studies emphasize structural differences in the depth, composition, and integration of venture capital markets. Compared to the US, Europe is characterized by a smaller number of large late-stage VC and growth equity funds, more fragmented capital markets, and a more limited participation of institutional investors such as pension funds and insurers in high-risk equity financing [16,17,23,27]. Cross-country analyses further stress the importance of IPO markets, market capitalization, and investor protection for VC activity, all of which tend to be more developed in the US context [23,40]. Policy-oriented studies by the European Commission, the European Investment Bank, and the European Investment Fund echo these findings, stressing that European VC ecosystems remain comparatively small, fragmented, and dependent on public actors despite extensive subsidy schemes [7,10–12]. This has led to the frequently used characterization of Europe as “subsidy-rich but capital-poor,” particularly in sectors dominated by intangible assets and high uncertainty [10,14].

On the demand side, the literature points to heterogeneity in firms’ scaling strategies, managerial capabilities, and international orientation. Quas et al. [9] argue that limited experience with rapid international expansion and organizational scaling may affect both firms’ demand for late-stage capital and investors’ perceptions of growth potential. Related work emphasizes that high-growth entrepreneurship is closely tied to managerial human capital, prior scaling experience, and access to global networks, factors that may differ systematically across regions [41,42].

Indirect evidence on the consequences of financing conditions is provided by research on firm relocation and cross-border investment. Weik et al. [43] show that foreign venture capital, particularly from the US, is strongly associated with startup relocation, with the majority of observed cross-border moves directed toward the United States. This pattern is consistent with the idea that access to deep and experienced VC markets matters for scaling.

Against this backdrop, artificial intelligence offers a particularly informative empirical setting. As a general-purpose technology characterized by rapid improvement, pervasiveness across sectors, and a strong reliance on complementary investments, AI places exceptional demands on scale-up financing [1,2,4]. Commercializing and scaling AI applications typically requires sustained access to large, risk-tolerant pools of capital, making AI-intensive ventures especially sensitive to the depth and flexibility of regional financial systems [3,27,29]. The post-2020 emergence of generative AI and large foundation models therefore provides a natural opportunity to examine whether a common technological shock translated into differential financial responses across regions, because it was around this time that AI moved from being a research-driven specialty into a dominant theme of mainstream venture investing. AI thus functions as a stress test of regional venture capital systems, allowing us to distinguish between differences in entrepreneurial pipelines and differences in scale-up finance.

### 2.4 Hypotheses

Based on the preceding conceptual background, we have derived three hypotheses about the evolution of AI-focused VC in Europe and the US.

First, if Europe’s AI “pipeline” is similar to that of the US, we should not observe a decline in AI startup formation. Instead, both regions should exhibit rising AI deal activity after the 2021 generative-AI inflection point. Given the global AI boom, we expect similar trajectories in AI deal counts. H1 (Deal activity – Pipeline): After Q2 2021, AI deal counts in Europe and the US increase substantially and follow parallel paths, implying no major transatlantic divergence in startup formation.

Second, if the US LME has a structural advantage in mobilizing large, risky pools of capital for frontier technologies, then we should observe a stronger VC reallocation toward AI in the US than in Europe. This should manifest as higher AI investment volumes and a larger share of total VC investment for AI. H2 (Investment volume – scale-up): Following the 2021 generative-AI inflection point, AI investment volumes grew more strongly in the US than in Europe.

Third, if the European scale-up financing gap is especially pronounced for capital-intensive, general-purpose technologies, then the transatlantic divergence should be particularly evident in AI investment intensity. While both regions may increase the relative weight of AI, the US is expected to reorient its VC portfolios more aggressively toward AI than Europe. H3 (AI investment intensity): The share of total VC volume represented by AI increases in both regions after Q2 2021, but more strongly in the US.

## 3 Data and empirical strategy

Our empirical strategy uses a DiD design. This design treats the 2021 generative-AI inflection point, the period around which large language models reached commercial maturity and AI moved from a research-driven niche to a dominant theme of mainstream venture investing, as a global shock to AI-related opportunities. Then, it compares the subsequent evolution of AI-focused VC in Europe and the US. Several assumptions are required for identification.

First, we assume that, in the absence of the post-2021 reallocation of venture capital toward AI, Europe and the US would have followed broadly parallel trends in AI venture activity. Figs 1 and 2 provide visual support for this assumption. Prior to Q2 2021, both regions’ AI deal counts and AI shares of total VC evolved in similar patterns. Section 4.5 reports a formal event study with year-by-region interaction coefficients (S1 Table). For our main intensity outcome (AI share of VC volume), the joint F-test of pre-treatment leads cannot reject the null of parallel trends (F = 1.20, p = 0.31). For log levels of AI volume and AI count, pre-treatment coefficients show a gradual narrowing of the EU-US gap, which is consistent with our argument that Europe was incrementally catching up before the 2021 reallocation. We discuss this more fully in Section 4.5.

**Fig 1:**
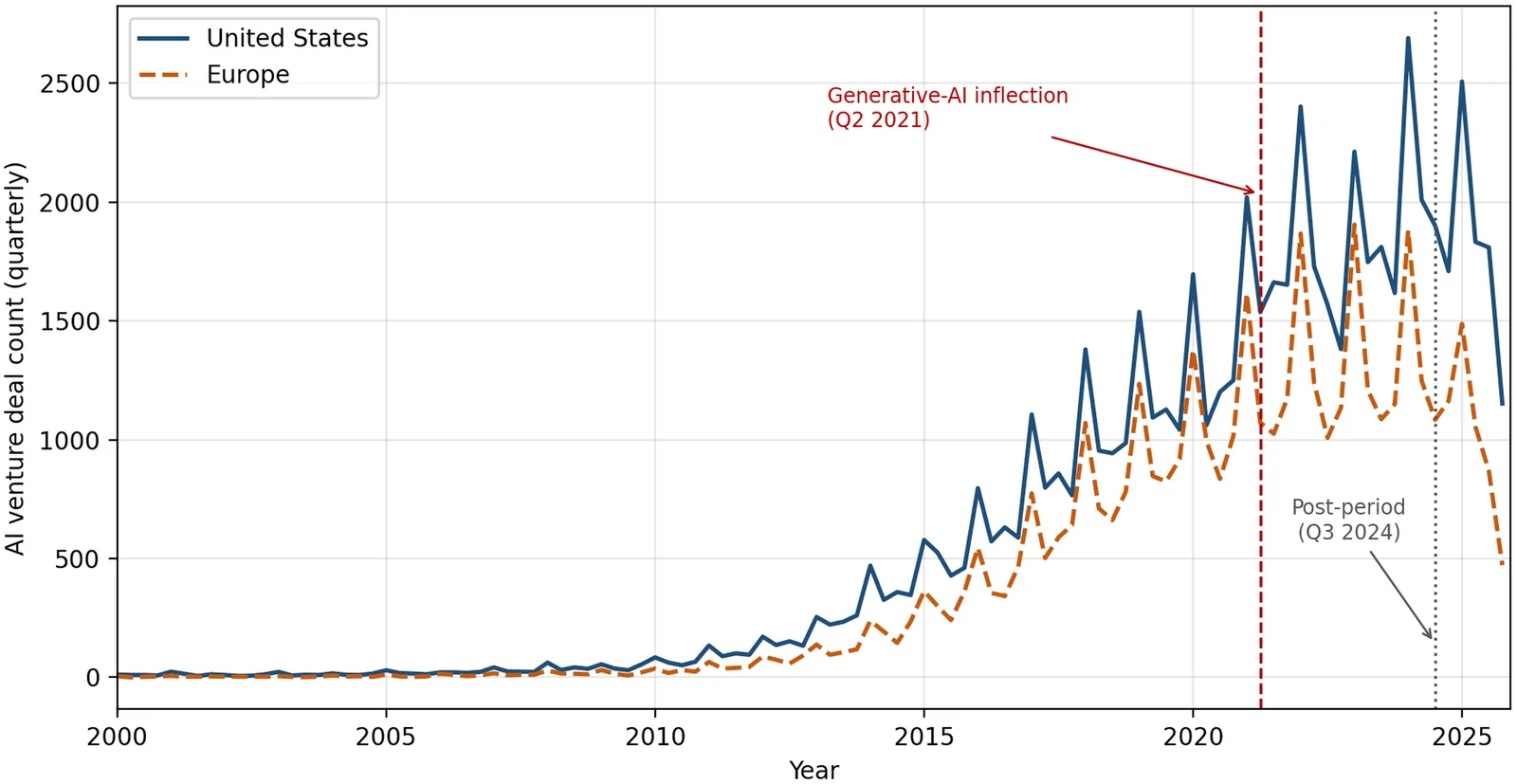
Quarterly AI venture deal count in Europe and the United States, 2000–2025. Vertical dashed lines indicate the start of the Anticipation phase (Q2 2021) and the Post-period (Q3 2024). The United States is shown as a solid line and Europe as a dashed line. The two breakpoints are labelled directly on the figure.

**Fig 2:**
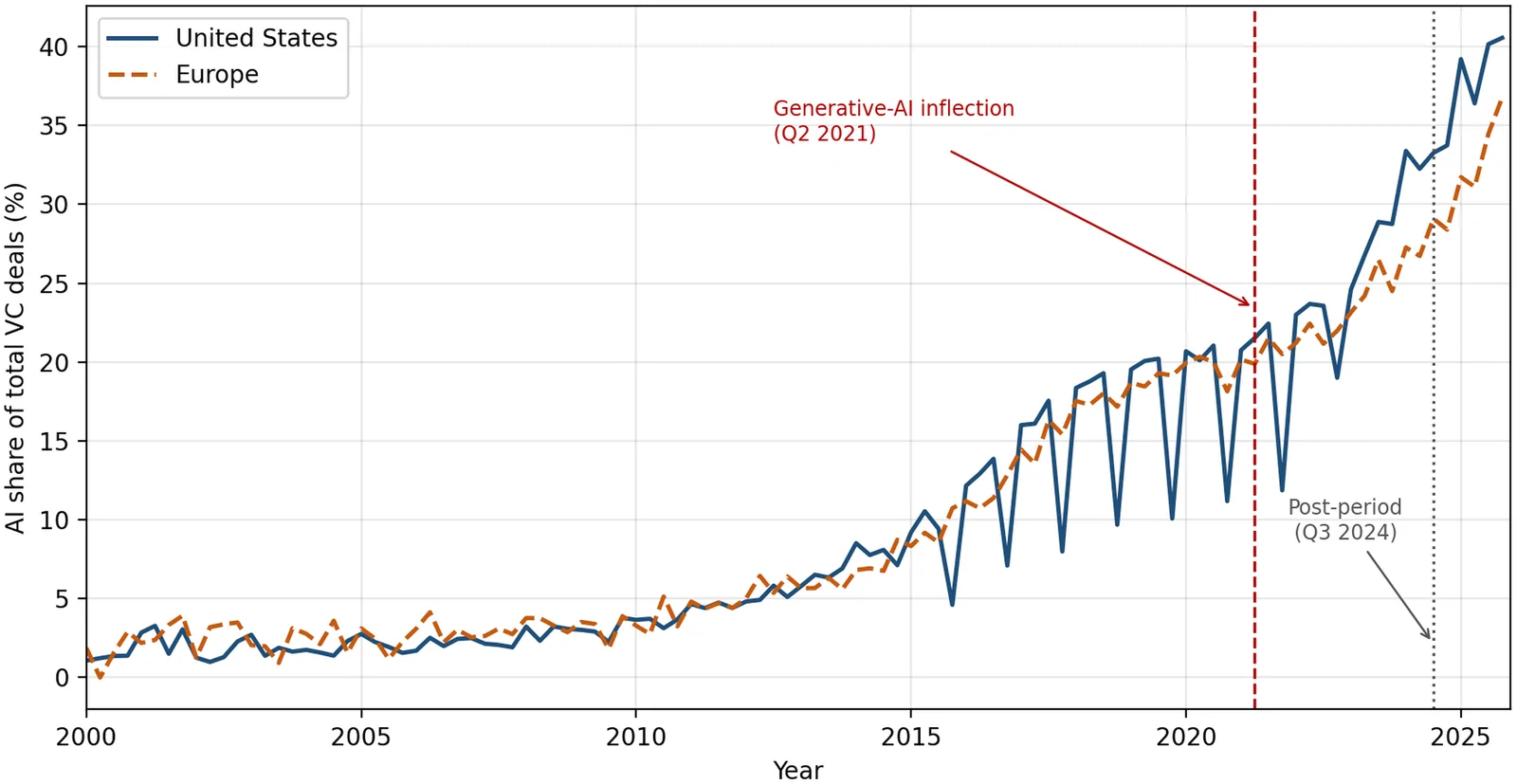
AI share of total venture deals in Europe and the United States, 2000–2025. Vertical dashed lines indicate the start of the Anticipation phase (Q2 2021) and the Post-period (Q3 2024). The United States is shown as a solid line and Europe as a dashed line. The two breakpoints are labelled directly on the figure.

Second, we assume that no other shock around 2021 affected AI VC in Europe and the US differentially in a way not captured by our control variables. To address concerns about confounding shocks, we incorporate macroeconomic control variables (e.g., GDP growth, inflation, unemployment, and population) and, in expanded models, include measures of innovation input and institutional quality (e.g., R&D expenditure, researchers per million inhabitants, rule of law, regulatory quality, and control of corruption). These variables absorb a wide range of business cycle, innovation, and governance differences that could otherwise bias the estimated treatment effects.

Third, we interpret the post-Q2 2021 coefficients as capturing the combined effect of the generative-AI commercialization shock and the subsequent AI boom, rather than a single, instantaneous treatment. Our anticipation and post-period dummies are designed to trace the buildup and consolidation of AI’s role in venture markets rather than to isolate a one-time shock.

Fourth, we do not attempt to identify a separate causal effect of any single regulatory measure. Our design captures the net outcome of technological and financial forces as they interact with the underlying varieties of capitalism in Europe and the US.

### 3.1 Data sources and sample construction

Our core dataset is drawn from PitchBook [44] and covers all venture-capital-backed financing rounds involving AI-related startups from Q1 2000 to Q4 2025. The extract was taken on 1 December 2025, so the final quarter (Q4 2025) is only partially observed; we assess the sensitivity of the results to this in a dedicated observed-window robustness check (S8 Table). We classify a startup as “AI-focused” if PitchBook tags its primary vertical as “Artificial Intelligence & Machine Learning.” Our analysis is restricted to deals with clearly identified headquarters in either the US or European countries with active VC markets, including the United Kingdom.

Three considerations support the validity and transparency of this PitchBook-based AI definition. First, PitchBook’s “Artificial Intelligence & Machine Learning” vertical is a curated tag applied by PitchBook analysts on the basis of company descriptions, product information, investor positioning and structured industry codes. The same tagging logic has been used by recent peer-reviewed analyses of AI venture capital, including Montanaro et al. [29] and the OECD’s 2025 AI VC review [4], which provides cross-source comparability. Second, any classification scheme based on company descriptors is subject to misclassification: AI-related activities embedded in broader software, fintech, or platform businesses may be missed, and some firms may be tagged as AI-focused on the basis of marketing claims that overstate the centrality of AI to their core product. Our identification strategy, however, is a difference-in-differences design that differences out any time-invariant misclassification that affects Europe and the US symmetrically. Misclassification would only bias our estimates if it shifted differentially across regions and around the Q2 2021 break, for which we find no a priori reason. Third, while granular cross-validation against an independent source (e.g., a stratified sample audited against Crunchbase tags or USPTO/EPO patent classifications) is beyond the scope of this paper, we acknowledge this as a limitation and flag the construction of a multi-source AI venture taxonomy as a productive avenue for future research (see Section 5.4). A second reason concerns how the tags are produced. PitchBook applies one taxonomy worldwide, with the same analyst team assigning verticals regardless of where a company is headquartered, so there is no separate European or U.S. tagging process that could raise the count for one region relative to the other. As with any classification error, such noise would affect the estimates only if it changed differentially around the Q2 2021 break.

For each region-quarter, we aggregate:

- the number of AI deals (ai_deal_count)
- total AI investment volume (ai_deal_volume)
- total VC deals and volumes (total_vc_deals and total_vc_volume).

Macroeconomic control variables, such as GDP growth, inflation, unemployment and population, as well as governance indicators such as rule of law, regulatory quality and control of corruption are obtained from the World Bank World Development Indicators and Worldwide Governance Indicators [45]. Innovation control variables such as R&D expenditure as a percentage of GDP and the number of researchers per million inhabitants, are obtained from the World Bank and OECD datasets [4,45].

We construct a panel indexed by region and time. To facilitate the DiD design, we divide the time dimension into three phases:

1. The Pre-period (Q1 2000–Q1 2021) covers the long build-up of AI research and early-stage venture activity, including the post-2012 deep-learning research boom but predating the commercial breakthrough of generative AI.
2. The Anticipation period (Q2 2021–Q2 2024) corresponds to the rapid commercialization of generative AI, the public release of large language models, and the surge of AI-focused VC fundraising.
3. The Post-period (Q3 2024–Q4 2025) is the phase in which AI becomes the dominant single theme in VC portfolios, allowing us to trace the consolidation of AI within venture allocations. We treat this boundary as a definitional split between the fast-growth and consolidation phases rather than as a second structural break; the anticipation and post dummies jointly capture the post-2021 reallocation, and the results do not hinge on the exact placement of the Q3 2024 cut.

Table 1 summarizes the number of region-time observations in each phase. In total, the panel comprises 208 region-time observations.

**Table 1:** Descriptive statistics for AI deal count by region and phase.

Phase	Number of obs.	Europe – Mean log AI deals	Europe – Sum of AI deals	US – Mean log AI deals	US – Sum of AI deals
Pre	170	3.83	20,568	4.58	29,007
Anticipation	26	7.15	16,997	7.50	24,017
Post	12	6.87	6,128	7.48	10,910
Total	208	–	43,693	–	63,934

Note: “Number of obs.” counts region-quarter observations per phase (the pre-period contains 170, i.e., 85 quarters for two regions). “Sum of AI deals” is the cumulative number of AI deals across all quarters in a phase and is therefore a total, not an average. The corresponding per-quarter averages are reported in Table 2.

The pre-period provides a long window in which to assess parallel trends, while the anticipation and post-periods allow us to trace the buildup and consolidation of the AI boom.

### 3.2 Variables and summary statistics

We analyze the levels and shares of AI activity. Our main outcomes are: log_ai_deal_count (the natural logarithm of the number of AI deals per region-quarter); log_ai_deal_volume (the natural logarithm of the total AI VC volume in USD millions); ai_share_count (the number of AI deals divided by the total number of VC deals); and ai_share_volume (the AI volume divided by the total VC volume).

The descriptive patterns already indicate three features. First, AI deal counts grow sharply in both regions across the Anticipation and Post phases. Second, AI investment volumes grow much faster in the US than in Europe. Third, AI’s share of total VC volume is roughly twice as high in the US as in Europe in the post-period (60% vs. 31%).

To visualize the underlying time trends, Fig 1 plots quarterly AI deal counts in Europe and the US. Fig 2 displays quarterly AI shares of total VC deals.

Both figures indicate that the trends before Q2 2021 are similar across regions. AI deal counts and AI shares grew slowly and together. After Q2 2021, both regions experienced a sharp increase, but the U.S. curve increasingly dominated, especially during the consolidation phase.

### 3.3 Difference-in-differences specification

To identify the impact of the generative-AI inflection point on regional AI VC dynamics, we employ a DiD design. Although the 2012 ImageNet breakthrough is widely regarded as the academic origin of the modern AI boom, the venture-capital reaction was concentrated after 2020, when generative AI became commercially salient and triggered a global surge in AI-focused VC fundraising [1,4]. We distinguish three periods: a pre-period (Q1 2000 to Q1 2021), an anticipation period (Q2 2021 to Q2 2024), and a post-period (Q3 2024 to Q4 2025). The anticipation and post dummies enter the specification as shown below, with the pre-period serving as the omitted base category.

Our baseline specification at the region-quarter level is given by Eq. (1):


$$\begin{array}{c} Y_{r,t}=\alpha +\beta_{1}\cdot Europe_{r}+\beta_{2}\cdot Anticipation_{t}+\beta_{3}\cdot Post_{t}+\beta_{4}(Europe_{r}\times Anticipation_{t})+\beta_{5}(Europe_{r}\times Post_{t})+\gamma \cdot X_{r,t}+\varepsilon_{r,t} \end{array}$$ (1)

where Y_{r,t} is one of our outcome variables for region r at time t. The coefficients of interest are the interaction terms, which capture how Europe’s trajectory differs from the US benchmark during the anticipation and post-periods. We rely on time-clustered standard errors to account for serial correlation across regions within the same quarter.

We estimate two sets of models: (i) simple DiD models without macroeconomic control variables to isolate the raw treatment effect; and (ii) extended models with macroeconomic control variables (business cycles, innovation inputs and institutional quality).

Rather than pinning this down to a single knife-edge quarter, we treat Q2 2021 as the onset of a broader reallocation window. Visual inspection of the underlying AI-share-of-VC-volume series shows no sharp, single-quarter jump but a gradual build-up concentrated in the years around 2019–2021, well before the mass-market ChatGPT inflection of Q4 2022, consistent with an investment-side treatment in which capital moved ahead of the consumer breakthrough. We therefore do not rely on a single date: we model the treatment as a window (an anticipation and a post-period), and S7 Table shows that using Q4 2022 as an alternative single break leaves the central result essentially unchanged (Europe × post on the AI volume share = −0.17, p < 0.001).

### 3.4 Robustness checks

We conducted several robustness checks. First, we estimate models with AI deal counts at different levels rather than in log form. Second, to ensure that mega-rounds do not drive the results, we re-estimate the log models after trimming the top 1% of region-quarter observations by AI deal volume. Third, to verify the parallel-trends assumption and the time pattern of treatment effects, we estimate a formal event study with year-by-region interactions and joint F-tests of pre-treatment leads (Section 4.5; S1 Table, S1 Fig). Fourth, we report a country-level concentration analysis of European VC and a sensitivity exercise that excludes the United Kingdom from the European aggregate (Section 5.4; Appendix Tables S2 and S3) to address intra-European heterogeneity. Fifth, we run a placebo test that assigns a false treatment year within the pre-treatment window (Section 4.5; S5 Table) and, sixth, we re-estimate the main models excluding the COVID-19 period 2020–2021 (Section 4.5; S6 Table). All six checks are cross-referenced from the results section and reported in full in the supporting Information.

## 4 Empirical results

In this section, we empirically evaluate the three hypotheses presented in Section 2.4. Hypothesis 1 (H1) examines the evolution of AI deal activity and explores whether Europe maintains similar levels of startup formation as the US following the Q2 2021 generative-AI inflection point. Hypothesis 2 (H2) focuses on AI investment volumes, testing whether the US experiences a greater increase in AI funding than Europe does. Hypothesis 3 (H3) focuses on AI investment intensity, examining whether AI absorbs a larger share of total VC funding in the US than in Europe since Q2 2021.

First, we present descriptive evidence, then turn to regression-based DiD estimates, and finally conduct robustness checks.

### 4.1 Descriptive patterns and the intensity gap

Tables 1 and 2, together with Figs 1 and 2, show that the AI boom is globally uneven. In both regions, average quarterly AI deal counts and AI shares rise steeply after Q2 2021. As Fig 1 shows, the deal-count trajectories of Europe and the US remain broadly parallel, especially during the anticipation phase; the summary statistics behind this are reported in Section 3.2 and are not repeated here.

**Table 2:** Average AI venture metrics by region and phase.

Region	Phase	AI deal count (quarterly avg.)	AI share of total deals	AI deal volume (USD m, quarterly avg.)	AI share of total VC volume
Europe	Pre	241.98	0.07	364.74	0.05
Europe	Anticipation	1,307.46	0.23	4,058.92	0.20
Europe	Post	1,021.33	0.32	4,435.60	0.31
United States	Pre	341.26	0.07	1,930.11	0.07
United States	Anticipation	1,847.46	0.25	18,309.38	0.32
United States	Post	1,818.33	0.37	42,218.72	0.60

Note: Deal counts and volumes are per-quarter averages within each phase. Multiplying a per-quarter average by the number of quarters in the phase reproduces the corresponding total in Table 1.

However, the pattern of investment volumes is quite different. European AI VC investment volume grows from approximately USD 365 million in the pre-period to around USD 4.4 billion in the post-period. In contrast, US AI VC investment volume jumps from USD 1.9 billion to over USD 42 billion (Table 2). Consequently, AI’s share of total VC volume reaches approximately 60% in the US but only 31% in Europe in the Post-period. This contrast should be read with two qualifications. First, total U.S. VC volume is several times larger than the European total, so the U.S. AI share is measured against a much larger base and the raw gap partly reflects the size of the U.S. market rather than AI orientation alone. Second, these shares are Post-period averages, whereas the divergence is tested formally by the difference-in-differences estimate on the AI volume share (Table 6, Panel B), where the Europe × post interaction is −0.26 (p < 0.001). We base our inference on that estimate rather than on the headline percentages. These descriptive statistics already suggest that Europe does not suffer from a shortage of AI startups. Rather, Europe struggles to fund them at scale.

### 4.2 Baseline regression results

Tables 3 and 4 present extended OLS models for log(AI deal count) and log(AI deal volume), including macroeconomic control variables. The specification reported here uses a parsimonious set of time-varying macroeconomic covariates (GDP growth, inflation, unemployment, R&D as a percentage of GDP). Region-invariant institutional indicators (rule of law, regulatory quality, control of corruption, log population) are absorbed by the is_eu indicator because they vary only between the US and the European aggregate. Including them simultaneously with is_eu produces a near-collinear specification. With the parsimonious specification, the is_eu indicator and its interactions are estimated on an economically interpretable scale. The R^2^ remains high (0.795 for count, 0.863 for volume).

**Table 3:** Baseline OLS for log(AI deal count) with macroeconomic control variables.

Variable	Coefficient	Std. error	t	p-value
Intercept	−9.18	1.35	−6.79	<0.001
is_eu	4.53	0.37	12.31	<0.001
anticipation	1.18	0.59	1.99	0.047
is_eu × anticipation	2.19	0.47	4.66	<0.001
post	2.58	0.15	17.14	<0.001
is_eu × post	0.22	0.13	1.73	0.083
gdp_growth	0.04	0.06	0.68	0.499
inflation	−0.47	0.06	−7.51	<0.001
unemployment	−0.26	0.05	−4.98	<0.001
rnd_gdp	5.91	0.43	13.68	<0.001

Note: Dependent variable: log_ai_deal_count (N = 208, R^2^ = 0.795). Standard errors clustered by quarter.

**Table 4:** Baseline OLS for log(AI deal volume) with macroeconomic control variables.

Variable	Coefficient	Std. error	t	p-value
Intercept	−8.42	1.23	−6.85	<0.001
is_eu	3.83	0.34	11.19	<0.001
anticipation	1.45	0.57	2.55	0.011
is_eu × anticipation	2.35	0.50	4.73	<0.001
post	>4.06	0.25	16.48	<0.001
is_eu × post	−0.14	0.20	−0.74	0.461
gdp_growth	0.08	0.05	1.46	0.145
inflation	−0.44	0.06	−7.63	<0.001
unemployment	−0.33	0.05	−6.78	<0.001
rnd_gdp	6.28	0.39	16.22	<0.001

Note: Dependent variable: log_ai_deal_volume (N = 208, R^2^ = 0.863). Standard errors clustered by quarter.

In the count model (Table 3), inflation and unemployment enter with intuitive negative signs and R&D intensity correlates strongly with AI deal counts. The post coefficient is large and highly significant (2.58), reflecting the global AI boom in the Post-period. The is_eu × post interaction is positive but only marginally significant (0.22, p = 0.08), indicating that Europe does not lag behind the US on the deal-count margin. This is consistent with the finding that startup formation is not the main source of divergence.

In the volume model (Table 4), the differences are more pronounced. The anticipation dummy is positive and significant (coefficient ≈ 1.45), reflecting the global increase in AI funding after Q2 2021. The post-dummy variable is substantial and highly significant (approximately 4.06), reflecting the sharp global increase in AI activity in the consolidation phase. The Europe interactions show that, after controlling for time-varying macro covariates, Europe is not behind the US on the count or volume margin within each phase. The negative is_eu × post interaction (−0.14, n.s.) does indicate a small relative under-performance of European AI volume in the most recent period, although it is not statistically distinguishable from zero in the volume model.

### 4.3 Difference-in-differences estimates

Table 5 presents simple DiD models that do not include macroeconomic control variables. For log(AI deal count), the post-period dummy is large and significant (2.91), indicating a strong global AI boom. The interaction term Europe × post is small (0.14) and only marginally significant (p ≈ 0.10). This suggests that Europe’s trajectory in deal counts does not diverge markedly from the US benchmark when we control for the general time shift.

**Table 5:** Simple DiD models (no macroeconomic control variables).

Variable	Coef. (Count)	SE	p	Coef. (Volume)	SE	p
Intercept	4.58	0.19	<0.001	6.07	0.20	<0.001
is_eu	−0.74	0.05	<0.001	−1.98	0.09	<0.001
anticipation	2.93	0.20	<0.001	3.67	0.23	<0.001
is_eu × anticipation	0.39	0.06	<0.001	0.53	0.14	<0.001
post	2.91	0.22	<0.001	4.50	0.27	<0.001
is_eu × post	0.14	0.08	0.097	−0.23	0.19	0.224

Note: Panel A: log_ai_deal_count (N = 208, R^2^ = 0.338). Panel B: log_ai_deal_volume (N = 208, R^2^ = 0.520).

In contrast, in the log(AI deal volume) model, the post-period dummy is larger (4.50), and the Europe × post interaction is negative (−0.23), though it is not statistically significant in this simple specification. When macroeconomic control variables are added, the pattern becomes clearer.

The DiD estimates reinforce the descriptive interpretation. The small Europe × post interaction in the count model is consistent with the finding that Europe’s trajectory in AI deal activity closely follows that of the US, in line with H1. The larger post-period effects for AI investment volumes and Europe’s weaker relative response indicate that the US translates the AI boom into substantially higher funding levels, supporting H2.

### 4.4 Artificial intelligence investment intensity

Table 6 focuses on AI investment intensity. In the ai_share_count model (Panel A), the post-period dummy is positive and highly significant (0.27), suggesting that AI ventures are becoming a central component of VC portfolios worldwide. The Europe main effect is negative but not statistically significant (−0.08, p = 0.13), so, conditional on the controls, Europe’s overall share of deals going to AI is close to the US level; the small but significant Europe × post interaction (−0.04, p < 0.001) indicates that a modest gap on this margin opens only after the treatment break. This deal-share gap is an order of magnitude smaller than the volume-share gap discussed below, and the deal-count models (Tables 3 and 5) show no divergence in the number of AI ventures.

**Table 6:** AI intensity models (share of AI in deals/volume).

Variable	Coef. (Count)	SE	p	Coef. (Volume)	SE	p
Intercept	−11.61	2.16	<0.001	−13.31	3.14	<0.001
is_eu	−0.08	0.05	0.125	−0.15	0.08	0.048
anticipation	0.09	0.03	<0.001	0.16	0.04	<0.001
is_eu × anticipation	0.04	0.02	0.008	−0.04	0.03	0.144
post	0.27	0.02	<0.001	0.50	0.05	<0.001
is_eu × post	−0.04	0.01	<0.001	−0.26	0.05	<0.001
gdp_growth	−0.00	0.00	0.493	−0.00	0.00	0.681
inflation	−0.01	0.00	<0.001	−0.01	0.00	0.004
unemployment	−0.01	0.00	<0.001	−0.01	0.00	<0.001
rnd_gdp	0.10	0.02	<0.001	0.09	0.03	0.009
log_population	0.59	y0.11	<0.001	0.68	0.16	<0.001

Note: Panel A: ai_share_count (N = 208, R^2^ = 0.892). Panel B: ai_share_volume (N = 208, R^2^ = 0.891).

The volume share model (Panel B) provides the strongest evidence of structural divergence. The post dummy is large and highly significant (0.50), indicating that after the treatment break, AI absorbs a much larger share of VC volumes. The Europe dummy is negative and significant (−0.15, p = 0.05), and the Europe × post interaction is also negative and highly significant (−0.26, p < 0.001). These regression results closely align with the descriptive statistics in Table 2 and the visual evidence in Fig 2. The US LME model is larger and more flexible in reallocating capital toward frontier technologies. Europe, by contrast, remains more diversified and less willing, or able, to concentrate risk capital in AI. These findings directly address H3.

### 4.5 Robustness

Table 7 presents a robustness check using AI deal counts in levels rather than logs. We estimate this model with the same parsimonious specification used in Tables 3 and 4, which omits the four region-invariant institutional indicators (rule of law, regulatory quality, control of corruption, and log population) because they are collinear with the is_eu indicator (see Section 4.2). The anticipation and post dummies are positive and highly significant, and the time-varying macroeconomic controls behave as in the log models. The is_eu × post interaction is negative and significant (−708.7, p < 0.001). In levels this reflects the much larger absolute base of U.S. deal activity, as the United States adds far more AI deals in raw counts. This is the level-based counterpart of the proportional (log) parallelism documented in Table 3 and does not overturn the deal-count finding.

**Table 7:** Robustness: AI deal count in levels (parsimonious specification).

Variable	Coefficient	Std. error	t	p-value
Intercept	−2,277.34	603.57	−3.77	<0.001
is_eu	1,175.07	159.15	7.38	<0.001
anticipation	966.52	249.08	3.88	<0.001
is_eu × anticipation	−126.70	136.12	−0.93	0.352
post	1,353.91	175.77	7.70	<0.001
is_eu × post	−708.73	61.35	−11.55	<0.001
gdp_growth	1.22	18.70	0.07	0.948
inflation	−73.00	25.89	−2.82	0.005
unemployment	−108.24	11.66	−9.28	<0.001
rnd_gdp	1,242.63	197.83	6.28	<0.001

Note: Dependent variable: ai_deal_count (N = 208, R^2^ = 0.838). Standard errors clustered by quarter. Region-invariant institutional indicators are omitted because they are collinear with is_eu (cf. Tables 3-4).

Table 8 reports results for the log(AI deal count) model after trimming the top 1% of AI deal-volume observations. This model uses the same parsimonious specification. The estimates are almost identical to the untrimmed baseline in Table 3. The is_eu × post interaction remains small and only marginally significant (0.33, p = 0.06), which indicates that the deal-count results are not driven by a handful of mega-rounds.

**Table 8:** Robustness: Log AI deal count, top 1% of deal-volume observations trimmed (parsimonious specification).

Variable	Coefficient	Std. error	t	p-value
Intercept	−9.17	1.35	−6.78	<0.001
is_eu	4.53	0.37	12.32	<0.001
anticipation	1.17	0.59	1.99	0.047
is_eu × anticipation	2.19	0.47	4.66	<0.001
post	2.47	0.15	15.98	<0.001
is_eu × post	0.33	0.18	1.88	0.060
gdp_growth	0.04	0.06	0.68	0.498
inflation	−0.47	0.06	−7.51	<0.001
unemployment	−0.26	0.05	−4.98	<0.001
rnd_gdp	5.91	0.43	13.68	<0.001

Note: Dependent variable: log_ai_deal_count (N = 205, R^2^ = 0.790). Top 1% of region-quarter observations by AI deal volume trimmed. Standard errors clustered by quarter.

To formally assess the parallel-trends assumption, we estimate an event-study specification with a separate Europe × Year interaction for each year between 2010 and 2025, with 2020 as the omitted reference year. The full set of coefficients is reported in S1 Table and visualized in S1 Fig. The leading question for parallel trends is whether the EU × Year interactions in the pre-treatment window (2010–2019) are jointly indistinguishable from zero. We report joint F-tests for three outcomes. (i) For our main intensity outcome, ai_share_volume, the joint F-test of pre-treatment leads cannot reject the null of parallel trends (F = 1.20, p = 0.31), supporting the validity of the DiD design for the share-based outcomes that drive our main scale-up finding. (ii) For log_ai_deal_volume, the joint test rejects strict parallelism (F = 2.68, p = 0.009), but inspection of the coefficient pattern (S1 Fig) shows a gradual narrowing of the EU-US gap from −0.49 (2010) to near-zero by 2017–2020, which is consistent with European AI VC incrementally catching up before the 2021 reallocation. (iii) For log_ai_deal_count, the joint test also rejects strict parallelism (F = 19.5, p < 0.001), again driven by a convergence pattern rather than divergence. The share-volume parallel-trends test is the most relevant for our scale-up argument. The level-based outcomes display a non-parallel “catching up” trend in the pre-period that is, if anything, conservative for our central conclusion that Europe diverges from the US on the volume-intensity margin in the Post-period.

As a further test of the design, we ran a placebo exercise that assigns a false treatment year within the pre-treatment window (2000–2020), when no generative-AI shock had occurred (S5 Table). For the two level-based outcomes, log AI volume and log AI deal count, the placebo Europe × false-post interaction is small and statistically insignificant for false breaks in both 2005 and 2010 (all p > 0.5), which is the result one would expect if the design were not generating a spurious effect. For the AI volume share, the placebo interaction is small (−0.036) but statistically significant. This is far smaller than the true post-period estimate (−0.26) and matches the event-study evidence that the two AI shares grew from near-zero levels at slightly different speeds over the long pre-period. Because it implies that Europe was already trailing marginally before the shock, it works against our central conclusion rather than in its favour. As an additional sensitivity check, we re-estimate the main models excluding the COVID-19 years 2020–2021 (S6 Table). The results are unchanged: the Europe × post interaction on the AI volume share remains negative and highly significant and is marginally larger in magnitude (−0.28, p < 0.001), which indicates that the intensity gap is not an artefact of pandemic-era financing conditions.

The robustness checks support the conclusions drawn from the baseline models. The main patterns are consistent with H1, H2 and H3, showing parallel growth in AI deal counts, a pronounced transatlantic divergence in AI investment volumes, and a stronger increase in AI investment intensity in the US.

## 5 Discussion

### 5.1 From pipeline problem to scale-up gap

The combined evidence from descriptive statistics, DiD models and robustness checks helps clarify the nature of the transatlantic divergence. The fact that AI deal counts in Europe and the US move in similar ways and that Europe’s AI deal share is only slightly lower than in the US, suggests that Europe does not have a “pipeline problem.” AI research excellence, entrepreneurial culture and early-stage funding appear sufficient to generate new ventures [12,13].

Instead, the divergence emerges at the scale-up stage, where the US ecosystem mobilizes far greater volumes of capital, allowing AI to dominate VC portfolios. This pattern has been documented in studies of the European scale-up finance gap [9] and is reflected in policy reports [10,11] and commentary [8,14,46].

Our findings therefore provide quantitative support for the idea that Europe is at risk of becoming an “incubator economy.” It generates promising AI startups but often fails to finance their global expansion, resulting in foreign acquisitions or relocations [43]. In GPT terms, Europe contributes substantially to AI invention and early experimentation but captures a smaller share of the downstream productivity and value creation.

The most informative feature of the results is the contrast between the two margins. Europe is at parity precisely where seed-stage policy operates, the generation of new AI ventures, and falls behind precisely where it does not, the supply of large, late-stage, risk-tolerant equity. The binding constraint is therefore the financing of AI firms at scale rather than their creation, and the two are easily conflated when only deal counts or only headline volumes are inspected. This has a direct interpretive consequence: instruments that add more early-stage deals act on a margin where Europe is already competitive, while the margin that binds, growth-stage capital, is left untouched. In general-purpose-technology terms, Europe helps invent AI and finances its early experimentation, but captures a smaller share of the capital-intensive complementary-investment phase in which most of the productivity gains from a GPT accrue; the relocation of scaling ventures toward deeper US capital markets [43] is the channel through which that value is exported. The pattern is thus less a story about weakness in European science or entrepreneurship than about the architecture of European capital markets.

### 5.2 Institutional Interpretation

The observed patterns are consistent with the comparative advantages and limitations of LMEs and CMEs. In the US LME context, deep stock markets, developed late-stage VC and growth equity funds, and flexible pension systems allow for the rapid redeployment of capital towards emerging technologies [16,23,27]. VC firms can raise substantial funds, syndicate mega-rounds, and exit through IPOs on liquid markets such as NASDAQ.

In the European CME context, however, the combination of bank-centric finance, fragmented capital markets and conservative institutional investors restricts the scale and speed of such reallocations. Even as policymakers promote AI strategies and “trustworthy AI” regulations, the underlying financial infrastructure remains incomplete. EIB [10] emphasizes that innovative European firms find it easier to obtain bank loans for tangible assets than to receive equity financing for intangible, AI-intensive projects. Arnold et al. [47] demonstrate that VC in Europe remains small relative to GDP and is susceptible to taxation and regulatory constraints.

Within-Europe evidence is consistent with the Varieties of Capitalism interpretation. Using country-level VC volumes 2021–2024 from PitchBook for 36 European economies and country-level institutional indicators from the World Bank Worldwide Governance Indicators and the World Development Indicators, European countries with stronger institutional foundations attract more private VC capital. The cross-country correlation between log VC volume 2021–2024 and (i) R&D as a percentage of GDP is 0.57; (ii) regulatory quality is 0.69; and (iii) control of corruption is 0.63 (S4 Table). These positive correlations align with the institutional channels emphasized by Hall and Soskice [15], Black and Gilson [27], and Grilli et al. [17] and offer suggestive evidence that the US-Europe scale-up gap reflects, in part, intra-European institutional differences that aggregate up to a continental capital-market deficit. Because country-level AI-specific deal data are not separately available in PitchBook’s public-licensed pivots, we frame this evidence as descriptive and consistent with, rather than a direct mechanism test of, the VoC interpretation. We read these correlations as descriptive support for an institutional-amplification mechanism, not as proof that differences in the variety of capitalism cause the observed transatlantic gap. One pattern is nonetheless suggestive. The United Kingdom, whose financial institutions sit closer to the liberal-market than to the coordinated-market type, accounts for the largest single national share of European VC (31.5%; S2 Table). Its position at the top of the European distribution fits the prediction that market-based institutions mobilise more risk capital, though here too we treat the evidence as indicative rather than decisive.

Our AI-specific results corroborate broader findings on the determinants of VC across countries [17,31]. Our results also align with the IMF’s assessment that Europe’s innovation paradox stems from a combination of fragmented markets, underdeveloped risk capital and regulatory complexity [8,47].

### 5.3 Policy implications

The empirical design identifies financing outcomes and does not evaluate any specific policy intervention. The directions outlined below should therefore be read as interpretive implications that are consistent with the observed scale-up gap, rather than as conclusions established directly by the analysis.

From a policy perspective, the findings suggest that increasing public seed funding or small thematic programs will not close the AI financing gap. The bottleneck lies in late-stage, large-scale, equity-based financing [9,47]. Three policy directions follow.

The most concrete implication follows directly from the deal-count versus volume contrast. Because Europe is already at parity in generating AI ventures, the marginal public euro is more effective at the growth stage than at the seed stage. This argues for shifting public support away from additional seed subsidies and accelerator programmes and toward growth-stage co-investment vehicles, for example a pan-European growth fund-of-funds and mandated co-investment alongside private leads on large (Series B and later) AI rounds, so that public capital is levered where the divergence actually occurs rather than where Europe already competes. The three broader directions set out below provide the enabling conditions for such vehicles to reach scale.

First, completing the Capital Markets Union (CMU) is critical. A genuine CMU would enable the creation of pan-European growth funds with sufficient scale to underwrite substantial AI investments, reduce the institutional investor’s home-bias, and improve exit conditions via deeper stock markets. Without such integration, even ambitious national initiatives will remain too small or fragmented to compete with US mega-funds [48,49].

Second, policymakers must unlock institutional capital. European pension funds and insurers manage trillions of euros but allocate only a small fraction to VC and growth equity, particularly domestically [10,11,50]. Regulatory frameworks could be recalibrated to reward long-term, diversified exposure to high-growth equity, especially in strategic domains such as AI and green technology.

Third, AI-specific regulations such as the EU AI Act should be designed to avoid exacerbating the scale-up gap. A risk-based approach to trustworthy AI is generally compatible with innovation [7,51], but compliance costs may disproportionately burden smaller firms and diminish Europe’s appeal as a location for scaling [52]. The EU AI Act should therefore be viewed as one element of a broader policy mix rather than a standalone instrument [29,52,53]: its effectiveness in narrowing the transatlantic AI divide depends on whether it is complemented by ambitious capital market reforms and measures that encourage private late-stage equity investment in AI. Without such complementary reforms, additional regulatory burdens may interact with shallow capital markets to delay AI adoption and weaken scaling dynamics, particularly for smaller firms whose adoption decisions are sensitive to perceived costs and the surrounding support environment [28,53].

### 5.4 Limitations and avenues for future research

Our analysis provides new evidence on the transatlantic dynamics of AI VC, but several limitations should be acknowledged.

First, our regional comparison is limited to Europe and the US. This focus is motivated by the prominence of these regions in the global AI debate and by data comparability. However, it necessarily leaves out other important AI hubs, such as China, Israel, and select Asian economies. Future work could expand the comparative framework to include more countries and investigate whether the scale-up financing gap observed in Europe is a general characteristic of CME or a phenomenon specific to certain regions.

Second, our measurement of “AI ventures” relies on PitchBook’s “Artificial Intelligence & Machine Learning” vertical tag (see Section 3.1). While these classifications are standard in the literature and in policy reports, they involve some inaccuracies and may overlook AI-related activities embedded in broader software or platform business models. Conversely, some firms may be tagged on the basis of marketing claims that overstate the centrality of AI to their core product. As discussed in Section 3.1, our DiD design differences out symmetric, time-invariant misclassification, but we acknowledge that constructing a multi-source AI venture taxonomy that triangulates PitchBook tags with Crunchbase classifications and patent-based AI indicators is a productive avenue for future work.

Third, we focus on VC outcomes, deal counts, investment volumes and AI investment intensity, rather than on downstream economic impacts. From a general-purpose technology perspective, the ultimate interest lies in how AI affects productivity, employment and inequality. A natural next step would be to link AI VC patterns to firm-level performance, sectoral productivity, or labor-market outcomes.

Fourth, the post-period is relatively short and, at its end, only partially observed: the venture data were extracted on 1 December 2025, so the final quarter (Q4 2025) captures an incomplete quarter. As a safeguard, S8 Table re-estimates the main models on fully observed windows ending in Q2 2025 and Q3 2025; the AI-volume-share divergence is essentially unchanged, so the central result does not depend on the most recent, partially observed quarter.

Fifth, we treat Europe as a relatively homogeneous entity in our DiD specification, though there is substantial heterogeneity within the continent. As S2 Table shows, the United Kingdom alone accounts for approximately 31.5% of total European VC volume between 2021 and 2024, followed by Germany (14.8%), France (13.7%), Sweden (6.6%) and the Netherlands (5.0%). To gauge the sensitivity of the European aggregate to UK inclusion, S3 Table reports a proportional-allocation sensitivity check: if the United Kingdom is removed from the European aggregate and Europe’s AI VC volume is rescaled by the country’s share of total European VC volume, the ratio of European AI VC to U.S. AI VC for 2021–2024 falls from 19.96% to 13.7%. The transatlantic gap is therefore even more pronounced once the largest single European VC ecosystem is excluded, supporting our central conclusion. Two limitations of this exercise apply. First, country-level AI-specific deal volumes for European countries are not separately available in our PitchBook pivots. We therefore approximate country-level AI activity by allocating Europe’s aggregate AI volume in proportion to country shares of Total VC volume. Second, this allocation assumes that AI intensity is uniform across European countries, which is itself an open empirical question. A natural next step would be to obtain country-level AI VC tags directly from PitchBook and re-estimate the DiD with country fixed effects, which would address both the heterogeneity concern and the demand-side question of whether the scale-up gap is concentrated in a subset of lagging European countries. The most valuable extension of this work, and the most direct test of the varieties-of-capitalism mechanism, would be a country-level difference-in-differences that treats the United Kingdom (a liberal-market economy) and coordinated-market economies such as Germany and France as separate units. Such an analysis requires country-level AI-tagged deal data that PitchBook does not currently license in disaggregated form. We therefore leave it for future work and, in the meantime, avoid reading too much into the continental aggregate.

Addressing these limitations would not only deepen our understanding of the financial foundations of AI innovation, but also provide targeted guidance for policymakers designing effective interventions.

## 6 Conclusion

This paper examined the evolution of AI-focused VC in Europe and the US between 2000 and 2025 through the frameworks of GPT diffusion and VoC. Using a DiD design around the Q2 2021 generative-AI inflection point and a large dataset of AI VC deals, we documented three main findings.

First, AI deal activity has surged globally, with Europe keeping pace with the US in terms of startup formation and AI’s share of total deals. Second, a pronounced divergence emerges in investment volumes and AI’s share of VC funding. In the Post-period, AI absorbs approximately 60% of VC investment in the US but only 31% in Europe. Third, this intensity gap persists after controlling for macroeconomic conditions, innovation inputs, and governance indicators. It also remains robust to alternative specifications, trimming of outliers, and a sensitivity check that excludes the United Kingdom from the European aggregate.

We interpret this divergence as evidence of a structural scale-up financing gap rather than a lack of European entrepreneurial capacity. The US LME system has a comparative advantage in mobilizing large pools of risk capital and redirecting them toward emerging GPTs, such as AI. By contrast, European CMEs remain constrained by bank-centric traditions, fragmented capital markets, and conservative institutional investors.

The policy implication is evident. Europe will not close the AI gap through minor adjustments or additional seed grants alone. Instead, Europe needs transformative reforms that deepen capital markets, unlock institutional capital for venture and growth equity, and ensure that efforts to finance AI scaling are commensurate with AI regulation. Most concretely, because the transatlantic gap is one of investment volume rather than deal count, the highest-leverage single intervention is to redirect public capital from seed subsidies toward growth-stage co-investment at the point where European ventures currently fail to scale. Future research could extend our analysis by incorporating deal-level data on valuations and investor syndicates, exploring heterogeneity across European subregions, and linking AI VC patterns to downstream productivity and employment outcomes.

Until these structural reforms are implemented, the frontiers of AI innovation and the economic benefits associated with GPT-driven growth will likely continue to favor the US.

## Supporting information

S1 Table Event-study estimates: Europe × Year interactions (reference year = 2020), with joint F-tests of pre-treatment interactions (2010–2019). Dependent variable: log_ai_deal_volume. Specification includes year fixed effects, an EU main effect, and Europe × Year interactions for 2010–2025 (omitting 2020 as reference); standard errors clustered by region-quarter. Joint F-tests for log_ai_deal_volume, log_ai_deal_count and ai_share_volume are reported (df_num = 10, df_denom = 96 in all three specifications).
(DOCX)

S2 Table Concentration of European VC volume by country, 2021–2024. Total European VC volume 2021–2024 ≈ USD 320 billion. Source: PitchBook (all VC stages, all sectors). UK = England + Scotland + Wales + Northern Ireland.
(DOCX)

S3 Table UK-exclusion sensitivity for the European AI VC aggregate, 2021–2024. The United Kingdom accounts for approximately 31.5% of total European VC volume 2021–2024 (see S2 Table). Because country-level AI-specific pivots are not separately available in our PitchBook data, the “Europe excluding UK” row is computed by allocating Europe’s aggregate AI volume proportionally to country shares of total European VC volume 2021–2024. This is a transparent illustrative proxy that assumes uniform AI intensity across European countries.
(DOCX)

S4 Table Cross-country correlations between log European VC volume 2021–2024 and institutional indicators (n = 36 European countries). Pairwise Pearson correlations across European countries with non-missing data. Country-level VC volume from PitchBook (2021–2024). Institutional indicators are 2018–2023 averages from World Bank WGI and WDI.
(DOCX)

S5 Table Placebo test: False treatment year within the pre-treatment window (2000–2020). Estimated on the pre-treatment window only (2000Q1–2020Q4), before any generative-AI shock, using the parsimonious specification. For the two level-based outcomes the placebo interaction is insignificant (p > 0.5). For the AI volume share it is small (−0.036) but significant, well below the true post-period estimate (−0.26, Table 6), and reflects a small pre-existing gap that makes our conclusion harder, not easier, to obtain.
(DOCX)

S6 Table Robustness: Main DiD estimates excluding the COVID-19 period (2020–2021). Observations for 2020 and 2021 are dropped and the parsimonious DiD is re-estimated. The Europe × post interaction on the AI volume share remains negative and highly significant and is slightly larger than in the full sample (−0.28 vs −0.26), confirming the intensity gap is not an artefact of pandemic-era financing.
(DOCX)

S7 Table Alternative treatment date: Single break at Q4 2022 (ChatGPT release). Difference-in-differences with a single treatment break at 2022Q4 (the public release of ChatGPT) instead of the Q2 2021 onset. Time-clustered standard errors; the pre-period is the omitted category. The Europe × post interaction on the AI volume share remains negative and highly significant (−0.17, p < 0.001), while the deal-count and volume interactions are positive, consistent with European deal-count parity. The central scale-up result does not depend on the exact break date.
(DOCX)

S8 Table Observed-window robustness: Main DiD estimates on fully observed quarters. The main models are re-estimated restricting the sample to fully observed quarters, dropping the partially observed final quarter (the PitchBook extract of 1 December 2025 captures an incomplete 2025Q4). The Europe × post interaction on the AI volume share remains negative and highly significant in every window (−0.23 to −0.25, p < 0.001), essentially unchanged from the full-sample estimate reported in Table 6.
(DOCX)

S1 Fig Event-study coefficients on Europe × Year interactions for log AI VC volume (reference year = 2020). Vertical dashed line: Q2 2021 treatment break. Error bars: 95% confidence intervals.
(TIF)

## References

[1] Calvino F, Haerle D, Liu S. Is generative AI a general purpose technology? Implications for productivity and policy. Paris: OECD Publishing. 2025. doi: 10.1787/704e2d12-en

[2] Bresnahan TF, Trajtenberg M. General purpose technologies ‘Engines of growth’?. Journal of Econometrics. 1995;65(1):83–108. doi: 10.1016/0304-4076(94)01598-t

[3] Bernstein S, Giroud X, Townsend RR. The Impact of Venture Capital Monitoring. The Journal of Finance. 2016;71(4):1591–622. doi: 10.1111/jofi.12370

[4] OECD. A sharp increase in AI-related venture capital investments could transform global economies and shape the future of artificial intelligence. Paris. 2025 [cited 2026 Sep 13]. Available from:https://oecd.ai/en/vc

[5] Broersma M. AI takes lion’s share of 2025 venture capital. Silicon UK. 2025 [cited 2026 September 13]. Available from: https://www.silicon.co.uk/e-enterprise/fund-raising/pitchbook-ai-vc-626862

[6] Dealroom. Europe Q2 2024 VC report. Amsterdam: Dealroom.co. 2024 [cited 2026 Sep 13]. Available from: https://dealroom.co/

[7] European Commission. Keeping European industry and science at the forefront of AI. 2025 [cited 2026 September 13]. Available from: https://commission.europa.eu/

[8] Ahmed H. Europe’s innovation paradox: sitting on a goldmine, yet starving for impact. 2024 [cited 2026 September 13]. Available from: https://oecdcogito.blog/2024/02/19/europes-innovation-paradox-sitting-on-a-goldmine-yet-starving-for-impact/

[9] Quas A, Mason C, Compañó R, Testa G, Gavigan JP. The scale-up finance gap in the EU: Causes, consequences, and policy solutions. Eur Manag J. 2022;40(5):645–52. doi: 10.1016/j.emj.2022.08.003 36465062 PMC9707731

[10] European Investment Bank. Investment report 2024/2025: innovation, integration and simplification in Europe. Luxembourg: European Investment Bank. 2025 [cited 2026 Sep 13]. Available from: https://www.eib.org/en/publications/20240354-investment-report-2024

[11] European Investment Fund. EU VC ecosystem – the VC Factor. Luxembourg: European Investment Fund. 2025 [cited 2026 September 13]. Available from: https://engage.eif.org/the-vc-factor/eu-vc-ecosystem

[12] Gabison GA. European start-up hotspots: an analysis based on VC-backed companies. Seville: European Commission, Joint Research Centre. 2016. Available from: https://publications.jrc.ec.europa.eu/repository/handle/JRC101215

[13] Merli A. Europe’s innovators are waking up. Finance & Development. 2025 [cited 2026 September 13]. Available from: https://www.imf.org/en/publications/fandd/issues/2025/06/europes-innovators-are-waking-up-alessandro-merli

[14] Serré L. The paradox of European start-ups: subsidy-rich, capital-poor. VentureLeap. 2025 [cited 2026 September 13]. Available from: https://www.ventureleap.io/

[15] Hall PA, Soskice D. Varieties of capitalism: the institutional foundations of comparative advantage. Oxford: Oxford University Press. 2001.

[16] Hege U, Palomino F, Schwienbacher A. Venture Capital Performance: The Disparity Between Europe and the United States. Finance. 2009;Vol. 30(1):7–50. doi: 10.3917/fina.301.0007

[17] Grilli L, Latifi G, Mrkajic B. Institutional determinants of venture capital activity: An empirically driven literature review and a research Agenda. Journal of Economic Surveys. 2019;33(4):1094–122. doi: 10.1111/joes.12319

[18] Kiranyaz S, Avci O, Abdeljaber O, Ince T, Gabbouj M, Inman DJ. 1D convolutional neural networks and applications: A survey. Mechanical Systems and Signal Processing. 2021;151:107398. doi: 10.1016/j.ymssp.2020.107398

[19] Murthy CB, Hashmi MF, Bokde ND, Geem ZW. Investigations of Object Detection in Images/Videos Using Various Deep Learning Techniques and Embedded Platforms—A Comprehensive Review. Applied Sciences. 2020;10(9):3280. doi: 10.3390/app10093280

[20] Krizhevsky A, Sutskever I, Hinton GE. ImageNet classification with deep convolutional neural networks. Advances in Neural Information Processing Systems. 2012;25:1097–105.

[21] Russakovsky O, Deng J, Su H, Krause J, Satheesh S, Ma S, et al. ImageNet Large Scale Visual Recognition Challenge. Int J Comput Vis. 2015;115(3):211–52. doi: 10.1007/s11263-015-0816-y

[22] LeCun Y, Bengio Y, Hinton G. Deep learning. Nature. 2015;521(7553):436–44. doi: 10.1038/nature14539 26017442

[23] Jeng LA, Wells PC. The determinants of venture capital funding: evidence across countries. Journal of Corporate Finance. 2000;6(3):241–89. doi: 10.1016/s0929-1199(00)00003-1

[24] Hall BH, Lerner J. The Financing of R&D and Innovation. Handbook of the Economics of Innovation. Elsevier. 2010. p. 609–39. doi: 10.1016/s0169-7218(10)01014-2

[25] Kerr WR, Nanda R. Financing Innovation. Annu Rev Financ Econ. 2015;7(1):445–62. doi: 10.1146/annurev-financial-111914-041825

[26] Carpenter RE, Petersen BC. Capital Market Imperfections, High‐Tech Investment, and New Equity Financing. The Economic Journal. 2002;112(477):F54–72. doi: 10.1111/1468-0297.00683

[27] Black BS, Gilson RJ. Venture capital and the structure of capital markets: banks versus stock markets. Journal of Financial Economics. 1998;47(3):243–77. doi: 10.1016/s0304-405x(97)00045-7

[28] Gupta V, Yang H. Study protocol for factors influencing the adoption of ChatGPT technology by startups. PLoS One. 2024;19(2):e0298427. doi: 10.1371/journal.pone.0298427 PMC10868733 38358993

[29] Montanaro B, Croce A, Ughetto E. Venture capital investments in artificial intelligence. J Evol Econ. 2024;34(1):1–28. doi: 10.1007/s00191-024-00857-7

[30] Hall PA, Gingerich DW. Varieties of Capitalism and Institutional Complementarities in the Political Economy: An Empirical Analysis. Brit J Polit Sci. 2009;39(3):449–82. doi: 10.1017/s0007123409000672

[31] Dimitrova L, Eswar SK. Capital Gains Tax, Venture Capital, and Innovation in Start-Ups. Review of Finance. 2022;27(4):1471–519. doi: 10.1093/rof/rfac057

[32] Allen F, Gale D. Comparing financial systems. Cambridge (MA): MIT Press. 2000.

[33] Kortum S, Lerner J. Assessing the Contribution of Venture Capital to Innovation. The RAND Journal of Economics. 2000;31(4):674–92. doi: 10.2307/2696354

[34] Hsu P-H, Tian X, Xu Y. Financial development and innovation: Cross-country evidence. Journal of Financial Economics. 2014;112(1):116–35. doi: 10.1016/j.jfineco.2013.12.002

[35] Fulton C, Mukherjee S. How Sweden became the Silicon Valley of Europe. Reuters. 2021 [cited 2026 September 13]. Available from: https://www.reuters.com/technology/how-sweden-became-silicon-valley-europe-2021-08-11/

[36] Zapoliansky K. 10 years that changed European VC: trends, sectors, and the road ahead. EU-Startups. 2025 [cited 2026 September 13]. Available from: https://www.eu-startups.com/2025/08/10-years-that-changed-european-vc-trends-sectors-and-the-road-ahead/

[37] Bottazzi L, Da Rin M. Venture capital in Europe and the financing of innovative companies. Economic Policy. 2002;17(34):229–70. doi: 10.1111/1468-0327.00088

[38] Da Rin M, Hellmann T, Puri M. A Survey of Venture Capital Research. Handbook of the Economics of Finance. Elsevier. 2013. p. 573–648. doi: 10.1016/b978-0-44-453594-8.00008-2

[39] Bertoni F, Colombo MG, Quas A. The patterns of venture capital investment in Europe. Small Bus Econ. 2015;45(3):543–60. doi: 10.1007/s11187-015-9662-0

[40] La Porta R, Lopez‐de‐Silanes F, Shleifer A, Vishny RW. Law and Finance. Journal of Political Economy. 1998;106(6):1113–55. doi: 10.1086/250042

[41] Cumming D, Werth JC, Zhang Y. Governance in entrepreneurial ecosystems: venture capitalists vs. technology parks. Small Bus Econ. 2019;52(2):455–84. doi: 10.1007/s11187-017-9955-6

[42] Colombo MG, Grilli L. On growth drivers of high-tech start-ups: Exploring the role of founders’ human capital and venture capital. Journal of Business Venturing. 2010;25(6):610–26. doi: 10.1016/j.jbusvent.2009.01.005

[43] Weik S, Achleitner A-K, Braun R. Venture capital and the international relocation of startups. Research Policy. 2024;53(7):105031. doi: 10.1016/j.respol.2024.105031

[44] PitchBook Data, Inc. PitchBook Platform. Seattle (WA): PitchBook Data, Inc. 2025.

[45] World Bank. World Development Indicators and Worldwide Governance Indicators. Washington (DC): World Bank. 2025.

[46] Menn A. Europe’s VCs must embrace risk. The Next Web. 2025 [cited 2026 September 13]. Available from: https://thenextweb.com/news/vcs-holding-back-european-ai-startups

[47] Arnold N, Claveres G, Frie JM. Stepping up venture capital to finance innovation in Europe. Washington (DC): International Monetary Fund. 2024. Available from: https://www.imf.org/en/publications/wp/issues/2024/07/10/stepping-up-venture-capital-to-finance-innovation-in-europe-551411

[48] Farrell J. Can the EU’s dual strategy of regulation and investment redefine AI leadership?. 2025 [cited 2026 September 13]. Available from: https://www.techpolicy.press/can-the-eus-dual-strategy-of-regulation-and-investment-redefine-ai-leadership/

[49] Belaey A. EU ramps up growth-stage start-up funding. Brussels: SwissCore. 2023 [cited 2026 September 13]. Available from: https://www.swisscore.org/

[50] Startup Savant. Countries with the biggest VC investment in 2023. TRUiC Startup Savant. 2024 [cited 2026 September 13]. Available from: https://startupsavant.com/

[51] Cajueiro DO, Celestino VRR. A comprehensive review of Artificial Intelligence regulation: Weighing ethical principles and innovation. Journal of Economy and Technology. 2025;4:77–91. doi: 10.1016/j.ject.2025.07.001

[52] Holst L, Lämmermann L, Mayer V, Urbach N, Wendt D. The impact of the EU AI Act’s transparency requirements on AI innovation. In: Wirtschaftsinformatik 2024 Proceedings, 2024. Available from: https://aisel.aisnet.org/wi2024/92/

[53] Ridzuan NN, Masri M, Anshari M, Fitriyani NL, Syafrudin M. AI in the Financial Sector: The Line between Innovation, Regulation and Ethical Responsibility. Information. 2024;15(8):432. doi: 10.3390/info15080432

