# Supplementary material for: Limited capital? Explaining transatlantic divergences in artificial intelligence startup financing
Source: PLoS One. 2026 Nov 5;21(11):e0342837. doi: 10.1371/journal.pone.0342837 (PMC13739153; doi:10.1371/journal.pone.0342837)
Supplement: Supplementary file 1 [file pone.0342837.s001.docx]

## S1 Table. Event-study estimates - Europe × Year interactions (reference year = 2020)

| Year | Coef. (EU × Year) | SE | p-value |
| --- | --- | --- | --- |
| 2010 | -0.485 | 0.242 | 0.045 |
| 2011 | -0.262 | 0.401 | 0.514 |
| 2012 | -0.359 | 0.316 | 0.256 |
| 2013 | -0.420 | 0.213 | 0.049 |
| 2014 | -0.613 | 0.272 | 0.024 |
| 2015 | -0.412 | 0.259 | 0.112 |
| 2016 | -0.283 | 0.115 | 0.014 |
| 2017 | 0.053 | 0.171 | 0.759 |
| 2018 | -0.004 | 0.126 | 0.972 |
| 2019 | -0.220 | 0.155 | 0.157 |
| 2020 (ref.) | 0.000 | - | - |
| 2021 | -0.165 | 0.132 | 0.209 |
| 2022 | 0.278 | 0.169 | 0.100 |
| 2023 | 0.067 | 0.318 | 0.833 |
| 2024 | -0.126 | 0.275 | 0.646 |
| 2025 | -0.788 | 0.204 | <0.001 |

*Note: Dependent variable: log_ai_deal_volume. Specification includes year fixed effects, an EU main effect, and Europe × Year interactions for 2010–2025 (omitting 2020 as reference). Standard errors clustered by region-quarter.*

*Joint F-tests of pre-treatment EU × Year interactions (years 2010–2019)*

| Outcome | F-stat | p-value | Interpretation |
| --- | --- | --- | --- |
| log_ai_deal_volume | 2.68 | 0.009 | rejects parallel trends, but pattern reflects gradual EU catch-up before 2021 |
| log_ai_deal_count | 19.5 | <0.001 | rejects parallel trends, convergence pattern, not divergence |
| ai_share_volume | 1.20 | 0.306 | fails to reject parallel trends; supports DiD identification for the main intensity outcome |

*df_num = 10, df_denom = 96 in all three specifications.*
