# Supplementary material for: Limited capital? Explaining transatlantic divergences in artificial intelligence startup financing
Source: PLoS One. 2026 Nov 5;21(11):e0342837. doi: 10.1371/journal.pone.0342837 (PMC13739153; doi:10.1371/journal.pone.0342837)
Supplement: Supplementary file 2 [file pone.0342837.s002.docx]

## S2 Table. Concentration of European VC volume by country, 2021–2024

| Country | Deal count | VC volume (USD m) | Share of EU VC volume (%) |
| --- | --- | --- | --- |
| United Kingdom | 17,955 | 100,716 | 31.5 |
| Germany | 4,352 | 47,322 | 14.8 |
| France | 6,401 | 43,755 | 13.7 |
| Sweden | 2,920 | 21,009 | 6.6 |
| Netherlands | 2,019 | 16,039 | 5.0 |
| Switzerland | 2,680 | 14,086 | 4.4 |
| Spain | 3,316 | 12,231 | 3.8 |
| Denmark | 2,179 | 7,447 | 2.3 |
| Italy | 2,411 | 7,128 | 2.2 |
| Finland | 1,277 | 6,356 | 2.0 |
| Belgium | 1,395 | 6,306 | 2.0 |
| Austria | 627 | 5,573 | 1.7 |
| Ireland | 1,297 | 5,505 | 1.7 |
| Norway | 1,048 | 5,056 | 1.6 |
| Estonia | 730 | 3,491 | 1.1 |

*Note: Total European VC volume 2021–2024 ≈ USD 320 billion. Source: PitchBook (all VC stages, all sectors). UK = England + Scotland + Wales + Northern Ireland.*
