# Supplementary material for: Limited capital? Explaining transatlantic divergences in artificial intelligence startup financing
Source: PLoS One. 2026 Nov 5;21(11):e0342837. doi: 10.1371/journal.pone.0342837 (PMC13739153; doi:10.1371/journal.pone.0342837)
Supplement: Supplementary file 3 [file pone.0342837.s003.docx]

## S3 Table. UK-exclusion sensitivity for the European AI VC aggregate, 2021–2024

| Aggregate | AI VC volume 2021–2024 (USD m) | Ratio to U.S. (%) |
| --- | --- | --- |
| Europe (full aggregate) | 64,127 | 19.96 |
| Europe excluding UK (proportional proxy) | 43,917 | 13.67 |
| United States | 321,263 | 100.00 |

*Note: The United Kingdom accounts for approximately 31.5% of total European VC volume 2021–2024 (see S2 Table). Because country-level AI-specific pivots are not separately available in our PitchBook data, the "Europe excluding UK" row is computed by allocating Europe's aggregate AI volume proportionally to country shares of Total European VC volume 2021–2024. This is a transparent illustrative proxy. It assumes uniform AI intensity across European countries.*
