# Supplementary material for: Limited capital? Explaining transatlantic divergences in artificial intelligence startup financing
Source: PLoS One. 2026 Nov 5;21(11):e0342837. doi: 10.1371/journal.pone.0342837 (PMC13739153; doi:10.1371/journal.pone.0342837)
Supplement: Supplementary file 4 [file pone.0342837.s004.docx]

## S4 Table. Cross-country correlations between log European VC volume 2021–2024 and institutional indicators (n = 36 European countries)

|  | log VC volume | R&D / GDP | Regulatory quality | GDP growth | Control of corruption |
| --- | --- | --- | --- | --- | --- |
| log VC volume | 1.00 | 0.57 | 0.69 | −0.22 | 0.63 |
| R&D / GDP | 0.57 | 1.00 | 0.78 | 0.05 | 0.76 |
| Regulatory quality | 0.69 | 0.78 | 1.00 | −0.10 | 0.92 |
| GDP growth | −0.22 | 0.05 | −0.10 | 1.00 | −0.07 |
| Control of corruption | 0.63 | 0.76 | 0.92 | −0.07 | 1.00 |

*Note: Pairwise Pearson correlations across European countries with non-missing data. Country-level VC volume from PitchBook (2021–2024). Institutional indicators are 2018–2023 averages from World Bank WGI and WDI.*
