# Supplementary material for: Limited capital? Explaining transatlantic divergences in artificial intelligence startup financing
Source: PLoS One. 2026 Nov 5;21(11):e0342837. doi: 10.1371/journal.pone.0342837 (PMC13739153; doi:10.1371/journal.pone.0342837)
Supplement: Supplementary file 5 [file pone.0342837.s005.docx]

## S5 Table. Placebo test - false treatment year within the pre-treatment window (2000–2020)

| Outcome | False treatment year | Europe × false-post coef. | SE | p-value | N |
| --- | --- | --- | --- | --- | --- |
| log AI deal volume | 2005 | 0.144 | 0.342 | 0.673 | 168 |
| log AI deal volume | 2010 | −0.142 | 0.227 | 0.531 | 168 |
| log AI deal count | 2005 | −0.036 | 0.176 | 0.838 | 168 |
| log AI deal count | 2010 | 0.021 | 0.138 | 0.878 | 168 |
| AI share of VC volume | 2005 | −0.036 | 0.008 | <0.001 | 168 |
| AI share of VC volume | 2010 | −0.042 | 0.008 | <0.001 | 168 |

*Note: Estimated on the pre-treatment window only (2000Q1–2020Q4), before any generative-AI shock, using the parsimonious specification. For the two level-based outcomes the placebo interaction is insignificant (p > 0.5). For the AI volume share it is small (−0.036) but significant, well below the true post-period estimate (−0.26, Table 6), and reflects a small pre-existing gap that makes our conclusion harder, not easier, to obtain.*
