# Supplementary material for: Limited capital? Explaining transatlantic divergences in artificial intelligence startup financing
Source: PLoS One. 2026 Nov 5;21(11):e0342837. doi: 10.1371/journal.pone.0342837 (PMC13739153; doi:10.1371/journal.pone.0342837)
Supplement: Supplementary file 6 [file pone.0342837.s006.docx]

## S6 Table. Robustness - main DiD estimates excluding the COVID-19 period (2020–2021)

| Outcome | Term | Coefficient | SE | p-value | N |
| --- | --- | --- | --- | --- | --- |
| log AI deal volume | Europe × post | 0.165 | 0.203 | 0.417 | 192 |
| log AI deal volume | post | 4.056 | 0.238 | <0.001 | 192 |
| AI share of VC volume | Europe × post | −0.281 | 0.047 | <0.001 | 192 |
| AI share of VC volume | post | 0.535 | 0.043 | <0.001 | 192 |
| log AI deal count | Europe × post | 0.557 | 0.129 | <0.001 | 192 |
| log AI deal count | post | 2.502 | 0.151 | <0.001 | 192 |

*Note: Observations for 2020 and 2021 are dropped and the parsimonious DiD is re-estimated. The Europe × post interaction on the AI volume share remains negative and highly significant and is slightly larger than in the full sample (−0.28 vs −0.26), confirming the intensity gap is not an artefact of pandemic-era financing.*
