# Supplementary material for: Limited capital? Explaining transatlantic divergences in artificial intelligence startup financing
Source: PLoS One. 2026 Nov 5;21(11):e0342837. doi: 10.1371/journal.pone.0342837 (PMC13739153; doi:10.1371/journal.pone.0342837)
Supplement: Supplementary file 7 [file pone.0342837.s007.docx]

## S7 Table. Alternative treatment date - single break at Q4 2022 (ChatGPT release)

| Variable | log AI deal volume | log AI deal count | AI share of VC volume |
| --- | --- | --- | --- |
| is_eu | 4.74 (p<0.001) | 5.32 (p<0.001) | 0.17 (p=0.199) |
| post (2022Q4) | 3.07 (p<0.001) | 2.32 (p<0.001) | 0.37 (p<0.001) |
| is_eu × post | 0.73 (p=0.109) | 0.80 (p=0.037) | −0.17 (p<0.001) |
| gdp_growth | 0.17 (p<0.001) | 0.11 (p<0.001) | 0.006 (p=0.001) |
| inflation | −0.34 (p<0.001) | −0.37 (p<0.001) | −0.008 (p=0.133) |
| unemployment | −0.30 (p<0.001) | −0.24 (p<0.001) | −0.006 (p=0.014) |
| rnd_gdp | 7.18 (p<0.001) | 6.67 (p<0.001) | 0.24 (p<0.001) |
| log_population | — | — | 0.05 (p=0.866) |
| N; R² | 208; 0.85 | 208; 0.80 | 208; 0.82 |

*Note: Difference-in-differences with a single treatment break at 2022Q4 (the public release of ChatGPT) instead of the Q2 2021 onset. Time-clustered standard errors; the pre-period is the omitted category. The Europe × post interaction on the AI volume share remains negative and highly significant (−0.17, p < 0.001), while the deal-count and volume interactions are positive, consistent with European deal-count parity. The central scale-up result does not depend on the exact break date. The positive is_eu main effect in this two-period specification is a conditional level difference and is not directly comparable to the phase-interacted models in the main text.*
