# Supplementary material for: Limited capital? Explaining transatlantic divergences in artificial intelligence startup financing
Source: PLoS One. 2026 Nov 5;21(11):e0342837. doi: 10.1371/journal.pone.0342837 (PMC13739153; doi:10.1371/journal.pone.0342837)
Supplement: Supplementary file 8 [file pone.0342837.s008.docx]

## S8 Table. Observed-window robustness - main DiD estimates on fully observed quarters

| Observation window | Outcome | Europe × post | SE | p-value | N |
| --- | --- | --- | --- | --- | --- |
| Through 2025Q3 (last complete) | AI share of VC volume | −0.233 | 0.047 | <0.001 | 206 |
| Through 2025Q3 | log AI deal volume | −0.095 | 0.213 | 0.655 | 206 |
| Through 2025Q3 | log AI deal count | 0.254 | 0.136 | 0.063 | 206 |
| Through 2025Q2 (reviewer) | AI share of VC volume | −0.249 | 0.056 | <0.001 | 204 |
| Through 2025Q2 | log AI deal volume | −0.207 | 0.216 | 0.339 | 204 |
| Through 2025Q2 | log AI deal count | 0.257 | 0.154 | 0.094 | 204 |

*Note: The main models are re-estimated restricting the sample to fully observed quarters, dropping the partially observed final quarter (the PitchBook extract of 1 December 2025 captures an incomplete 2025Q4). The Europe × post interaction on the AI volume share remains negative and highly significant in every window (−0.23 to −0.25, p < 0.001), essentially unchanged from the full-sample estimate reported in Table 6. The scale-up finding is not an artefact of the short or partially observed post-period.*
